# Recent Advances in Plant-Based Emulsion Gels: Preparation, Characterization, Applications, and Future Perspectives

**DOI:** 10.3390/gels11080641

**Published:** 2025-08-13

**Authors:** Yunfei Huang, Chunmei Li, David Julian McClements

**Affiliations:** 1 Department of Food Science, University of Massachusetts Amherst, Amherst, MA 01003, USA; yunfeihuang@umass.edu; 2 College of Food Science and Technology, Huazhong Agricultural University, Wuhan 430070, China; lichmyl@mail.hzau.edu.cn

**Keywords:** plant-based, emulsion gel, food application, characterization

## Abstract

Plant-based foods have emerged as a major focus of the modern food industry as it tries to create more sustainable, environmentally friendly, and healthy products. Plant-based emulsion gels (PBEGs) can be used to provide valuable structures, textures, and functions in many plant-based food applications. For instance, they can be used as a matrix to form semi-solid plant-based meat, fish, egg, or dairy analogs, delivery systems for bioactive compounds in functional foods, and edible inks in 3D food printing. The most common PBEGs used in the food industry consist of oil droplets embedded within an aqueous phase containing a biopolymer network. However, PBEGs may also be formed from high-internal-phase emulsions (HIPEs) or aggregated emulsions. PBEGs combine the benefits of emulsions and gels, such as the ability to encapsulate both polar and non-polar functional ingredients, as well as to create desirable textural attributes. This review summarizes recent advances (2017–2025) in the development and application of PBEGs in the food sector, with a focus on their preparation methods, characterization techniques, and potential applications. The future perspectives and challenges associated with PBEGs are also discussed. Overall, this review provides a useful platform for directing future research efforts and for the practical implementation of PBEGs in plant-based food systems.

## 1. Introduction

Emulsion gels are soft solid materials that combine the advantages of both emulsions and gels [1]. Emulsion gels can have semi-solid characteristics for several different reasons, including the presence of a polymer matrix in the aqueous phase, the aggregation of the droplets, or the close packing of the droplets [2]. In response to concerns about the negative environmental impacts of raising animals for food, there has been growing interest in pursuing alternative sources of protein-rich foods that are nutritious, environmentally friendly, and sustainable [3]. Among these, plant-based foods, such as meat, fish, egg, and dairy analogs, have become a key area of innovation in the modern food industry [4]. Plant-based emulsion gels (PBEGs) can be used as functional ingredients in the formulation of plant-based foods, as they can provide novel textural and structural properties, as well as serving as carriers for a broad range of functional ingredients, including colors, flavors, nutrients, and nutraceuticals. In this article, we primarily focus on oil-in-water (O/W) emulsion gels because they are the most commonly used in the food industry.

**Figure 1:**
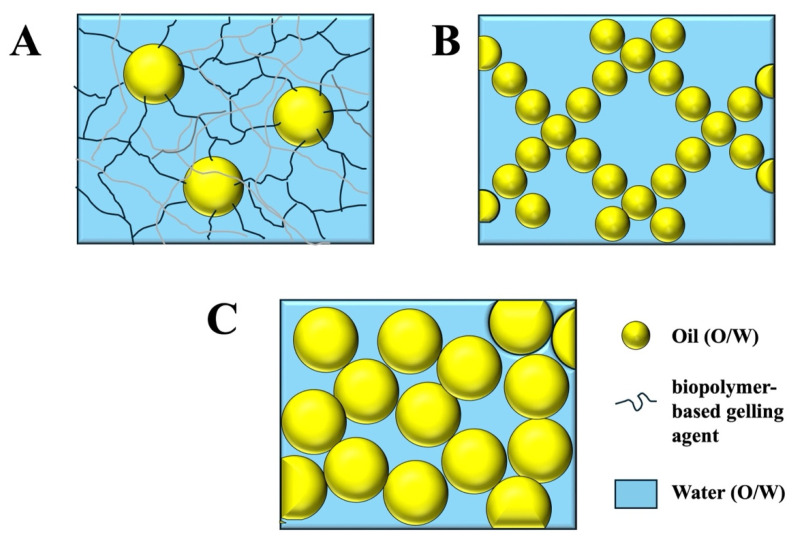
Illustration of the structure of the three main kinds of emulsion gels: (**A**) polymer-gelled emulsions; (**B**) aggregated emulsions; (**C**) jammed emulsions. Typically, the size of the oil droplets in emulsion gels depends on the method used to prepare the emulsions and may vary from around 100 nm to 100 μm.

In general, there are three main types of emulsion gels, which gain their semi-solid properties through different mechanisms (Figure 1), as follows:

(i) Polymer-gelled emulsions: This type of emulsion gel consists of oil droplets dispersed within a gelled aqueous phase. Typically, the aqueous phase is gelled using an appropriate biopolymer-based gelling agent, such as a protein and/or polysaccharide. The elastic properties of this type of system are, therefore, mainly determined by the nature of the gel in the aqueous phase.
(ii) Aggregated emulsions: this type of emulsion gel contains a 3D network of aggregated oil droplets that extends throughout the entire volume of the system, thereby providing elastic properties.
(iii) Jammed emulsions: In this type of emulsion gel, the droplet concentration is so high that the droplets are jammed tightly together, and so they cannot easily move past each other when the sample is deformed. The elastic properties of this type of gel are, therefore, a result of the resistance of the tightly packed oil droplets to move. High-internal-phase emulsions (HIPEs) are an example of this kind of emulsion gel.

In the remainder of this article, we mainly focus on polymer-gelled emulsions, as these have the greatest range of applications; however, we also mention other types where appropriate. Polymer-gelled emulsions can be further classified based on the nature of the gelling agent within the aqueous phase, i.e., as protein-, polysaccharide-, and protein/polysaccharide-based systems [5]. Commonly used plant proteins in food applications include soy, pea, and potato protein, while commonly used polysaccharides include agar, alginate, carrageenan, and pectin [6]. The type of matrix material and the gelation strategy employed determine the stability, mechanical properties, processing adaptability, and functional performance of PBEGs in food systems. For this reason, we provide an overview of the different approaches to create protein-, polysaccharide-, and protein/polysaccharide-based emulsion gels.

The successful development and application of PBEGs within the food industry requires knowledge of their structure–function relationships. A range of analytical techniques are typically required to characterize the structural, physicochemical, and functional properties of emulsion gels, including microscopy, rheology, particle size, interfacial charge, and thermal stability [7]. For this reason, a brief overview of the different analytical methods commonly used to characterize the properties of emulsion gels is also given.

Knowledge of the properties of emulsion gels is important for designing them for specific applications. In general, PBEGs have considerable promise as functional materials in a broad range of applications within the food industry. A literature search was conducted using PubMed and Web of Science for studies published between 2017 and 2025, focusing on the formulation, characterization, and application of PBEGs in food systems. Based on this literature review, we provide a brief overview of several applications of emulsion gels in foods that have already been explored, including as fat replacers, meat analogs, delivery systems, edible inks, and in baked goods. Finally, future perspectives and current challenges to the implementation of PBEGs in the food industry are discussed.

## 2. Preparation of Plant-Based Emulsion Gels

The aqueous phase of polymer-gelled emulsions can be gelled using proteins and/or polysaccharides that can form physical or chemical crosslinks with each other [8]. In this section, we therefore review the properties of protein-, polysaccharide-, and protein/polysaccharide-based emulsion gels. A summary of different preparation methods used to prepare plant-based emulsion gels in previous studies is shown in Table 1.

### 2.1. Protein-Based Emulsion Gels

The amphiphilic properties of proteins means they are also effective emulsifiers, capable of adsorbing at oil–water interfaces to form and stabilize oil-in-water emulsions [7]. Moreover, many plant proteins can form hydrogels in aqueous solutions under appropriate gelation conditions [9]. Owing to these attributes, plant proteins can be used as dual-function ingredients in PBEGs, where they can function as emulsifiers to form and stabilize the oil droplets, as well as gelling agents, to form the hydrogel in the aqueous phase. Proteins can be made into gel through a variety of mechanisms, including heat-, pH-, salt-, and enzyme-set mechanisms [5], which allows the properties of emulsion gels to be tailored for different applications. In the remainder of this section, the most commonly used protein gelation mechanisms are briefly reviewed.

#### 2.1.1. Heat-Set Gelation

Heat-set gelation is one of the most common strategies for preparing protein-based emulsion gels [22]. Initially, an oil-in-water emulsion is prepared that contains native globular proteins in the aqueous phase and/or around the surfaces of the oil droplets. A wide variety of globular plant proteins can be used for this purpose, including those isolated from soybeans, peas, or potatoes. Upon heating above their thermal denaturation temperature, these globular proteins unfold and expose non-polar and sulfur-containing amino acid side groups, thereby facilitating protein–protein interactions through hydrophobic attraction and disulfide bond formation [23]. At sufficiently high protein or droplet concentrations, these interactions lead to the formation of a 3D network that traps the oil droplets, resulting in the formation of an emulsion gel [24]. As a specific example, researchers have created heat-set emulsion gels by heating potato-protein-stabilized emulsions containing free potato protein in the aqueous phase [25]. Similarly, Teng et al. [26] prepared heat-set emulsion gels by incubating pea-protein-stabilized oil-in-water emulsions in a water bath at 70 °C, followed by rapid cooling.

#### 2.1.2. Salt-Set Gelation

Salt-set gelation can also be used to form emulsion gels by promoting the aggregation of proteins and/or protein-coated oil droplets in emulsions by modulating the electrostatic interactions in the system [27]. Typically, an oil-in-water emulsion is first formed that contains free proteins in the aqueous phase and/or protein-coated oil droplets. Then, oppositely charged salt ions (counter-ions) are added to the emulsion. For instance, if the proteins have a negative charge (pH > isoelectric point), then positively charged counter-ions (such as Na^+^, K^+^, Ca^2+^, or Mg^2+^) can be added. These ions can reduce the electrostatic repulsion between the protein molecules and/or protein-coated oil droplets through electrostatic screening and ion-binding effects, which can promote droplet aggregation [9]. In the case of multivalent counter-ions, they can also promote droplet aggregation by forming electrostatic salt bridges between the proteins. At sufficiently high protein or droplet concentrations, a 3D gel network is formed that is typically held together by a combination of van der Waals attraction, hydrophobic attraction, and salt bridge formation [28]. The type and concentration of salt ions added influences the gelation process and microstructure formed, which impacts the mechanical strength, fluid-holding properties, and stability of the final emulsion gel [29]. As a specific example, researchers have formed salt-set emulsion gels by adding calcium ions to soy-protein-stabilized emulsions under neutral pH conditions, where the proteins have a net negative charge [10]. Similarly, Zhao et al. [30] found that the addition of low concentrations of sodium chloride enhanced the gel strength of soy-protein-based emulsion gels, whereas high concentrations had an inhibitory effect.

#### 2.1.3. pH-Set Gelation

Protein-based emulsion gels can also be formed by adjusting the pH to a particular value that promotes protein aggregation [31]. Again, an oil-in-water emulsion is typically formed that contains free proteins in the aqueous phase and/or protein-coated oil droplets. Then, the acids or bases are added to the emulsion to adjust its pH close to the isoelectric point (pI) of the proteins. At or near this point, the net charge on the protein molecules is close to zero, thereby weakening the electrostatic repulsion between the proteins and protein-coated oil droplets in the emulsion, which usually promotes aggregation through a combination of van der Waals attraction, hydrophobic attraction, and hydrogen bonding [32]. At a sufficiently high concentration, protein and/or droplet aggregation leads to the formation of a three-dimensional network, resulting in viscoelastic gels. As an example, researchers have formed pH-set emulsion gels by acidifying soy-protein-stabilized oil-in-water emulsions [11]. Lin et al. [33] found that pH significantly affected the gelation of soy-protein-based emulsion gels, which was mainly attributed to alterations in the magnitude of the electrostatic repulsion between the protein-coated oil droplets. At pH 7.0, the droplets tended to flocculate with each other, leading to the formation of an emulsion gel. Moreover, the gel strength could be modulated by varying the pH.

#### 2.1.4. Enzyme-Set Gelation

Protein-based emulsion gels can also be formed by adding food-grade enzymes capable of covalently crosslinking the proteins such as transglutaminase (TGase) and laccase [34]. For example, TGase catalyzes the formation of ε-(γ-glutamyl)-lysine bonds between glutamine and lysine residues [12]. In contrast, laccase oxidizes certain amino acid groups (mainly tyrosine), which generates phenoxy radicals that can dimerize or react with other amino acid residues, thereby creating protein–protein crosslinks [35]. Initially, an oil-in-water emulsion is again formed that contains protein-coated oil droplets and/or free proteins in the aqueous phase. Then, an appropriate amount of enzyme is added to the aqueous phase and the system is incubated under optimum conditions for enzyme activity (such as pH and temperature). Compared to heat- or acid-set methods, enzyme-set gelation typically occurs at ambient or physiological temperatures, preserving the functionality of sensitive compounds and allowing for precise control over gelation kinetics. As an example, researchers have prepared pea-protein-based emulsion gels using TGase as a crosslinking agent [12]. Similarly, Zhang et al. [36] prepared soy-protein-based emulsion gels using laccase- and transglutaminase-induced methods.

#### 2.1.5. Chemical-Set Emulsion Gels

Protein-based emulsion gels can also be created using specific chemicals that can covalently crosslink proteins, such as genipin or glutaraldehyde [13]. Initially, an oil-in-water emulsion is formed containing protein-coated oil droplets and/or free proteins, and then an appropriate amount of the crosslinking chemical is added. The reaction mixture is then incubated under appropriate conditions (such as pH, temperature, relative humidity, oxygen levels, or light exposure). The covalent crosslinks formed enhanced the mechanical strength, thermal stability, and enzymatic resistance of the resulting emulsion gels, which is useful for some food applications, such as fruit coatings and the delivery of bioactive compounds [37,38]. Despite their advantages, the use of chemical crosslinkers in food systems requires careful consideration of potential toxicity, residual reagents, and regulatory approval. Therefore, natural, food-grade crosslinkers are usually preferred.

### 2.2. Polysaccharide-Based Emulsion Gels

Polysaccharides can also be used as gelling agents in emulsion gels [39]. In this case, gelation is usually a result of the physical or chemical crosslinking of polysaccharide molecules in the aqueous phase of oil-in-water emulsions [40]. Like proteins, a range of different strategies can be employed to induce the gelation of emulsions containing polysaccharides, including heat-, cold-, salt-, pH-, and enzyme-set methods. The polysaccharides used for this purpose may be derived from plants (such as starch, cellulose, and pectin) seaweed (such as carrageenan and alginate), or microbial fermentation (such as gellan gum) [41]. For the sake of concision, all these different sources of polysaccharides will be referred to as non-animal-based.

#### 2.2.1. Heat-Set Gelation

Emulsion gels can be formed by adding polysaccharides capable of forming gels when they are heated, such as starch [40]. In this case, starch granules are dispersed within the aqueous phase of an oil-in-water emulsion at ambient temperature. The emulsion is then heated above the gelatinization temperature of the starch, which causes the starch granules to absorb water and swell. At a sufficiently high concentration, the swelling of the starch granules causes them to become closely packed together, which leads to the formation of a hydrogel in the aqueous phase. In addition, the starch granules may become disrupted, releasing starch molecules. During cooling, these molecules undergo a coil-to-helix transition and then hydrogen bonds are formed between the helices, leading to the formation of crosslinks. Methylcellulose can also form heat-set gels, but these tend to weaken their structure when the samples are cooled below a particular temperature [42].

#### 2.2.2. Cold-Set Gelation

Certain plant-based polysaccharides, such as agar and gellan gum, form gels when they are cooled [42]. In other words, these polysaccharides form cold-setting thermoreversible hydrogels. Initially, an oil-in-water emulsion is formed and then a cold-setting polysaccharide ingredient is added to the aqueous phase. The emulsion is then heated above the helix-coil transition temperature of the polysaccharide molecules, which causes them to adopt a random coil conformation. Subsequently, the emulsion is cooled below this transition temperature, which causes helical regions to form on the polysaccharide molecules. At a sufficiently high concentration, these helical regions form crosslinks with helical regions on other molecules, leading to the formation of a hydrogel in the aqueous phase. These kinds of emulsion gels can often remain stable over a wide range of pH and ionic conditions [43]. As an example of this approach, researchers have created emulsion gels by adding agar to an oil-in-water emulsion, followed by heating and cooling to induce cold-set gelation [14].

#### 2.2.3. Salt-Set Gelation

Salt-set gelation is commonly used to form polysaccharide-based emulsion gels [44]. Anionic polysaccharides, such as agar, alginate, pectin, and carrageenan, are often used to form these kinds of hydrogels. In this approach, monovalent or multivalent cations (e.g., K^+^, Ca^2+^, Ba^2+^, or Zn^2+^) are used to induce gelation by forming salt bridges between anionic functional groups (such as carboxyl or sulfate groups) on the polysaccharide molecules [45]. Typically, an oil-in-water emulsion is formed and then an ionic polysaccharide is added to the aqueous phase. Then, an appropriate counter-ion is added to induce crosslinking of the polysaccharide molecules, leading to the formation of a hydrogel in the aqueous phase that traps the oil droplets. In some cases, it is important to slowly release the counter ions to produce a more homogeneous hydrogel structure [46]. This can be achieved by using a slowly dissolving salt, such as calcium carbonate during acidification with glucono-delta-lactone (GDL) [47]. In contrast, simply mixing a counter-ion solution with the emulsion can lead to the formation of a highly heterogeneous structure, often referred to as “fisheyes” because large gelatinous clumps are formed. The most common example of salt-set gelation is the addition of calcium ions to an emulsion containing sodium alginate [48].

#### 2.2.4. pH-Set Gelation

Many polysaccharides are relatively insensitive to changes in pH in terms of their gelation properties [49]. However, there are some examples where polysaccharides can be made to form gels by changing the pH. For instance, high-methoxy pectin will form acid-set gels in the presence of relatively high concentrations of sugar [50]. In this case, emulsion gels could be formed by incorporating a sufficient amount of pectin and sugar within the aqueous phase of an oil-in-water emulsion under neutral conditions and then acidifying the aqueous phase.

#### 2.2.5. Enzyme-Set Gelation

Some polysaccharides can be crosslinked by specific enzymes, which enables enzyme-set polysaccharide-based emulsion gels to be formed. For instance, feruloylated arabinoxylans can participate in oxidative coupling reactions catalyzed by enzymes like laccase or peroxidase, which leads to the formation of polysaccharide–polysaccharide crosslinks, thereby forming hydrogels [51]. As an example, researchers formed emulsion gels by adding peroxidase to catalyze the crosslinking of corn fiber gum in the aqueous phase of an oil-in-water emulsion [17].

### 2.3. Protein/Polysaccharide-Based Emulsion Gels

Composite emulsion gels with new or improved properties can often be created by combining proteins and polysaccharides together. For instance, amphiphilic proteins can be used to stabilize oil-in-water emulsions, while polysaccharides can be used to induce gelation of the aqueous phase [52]. Alternatively, both proteins and polysaccharides can be used to gel the aqueous phase [53]. Different kinds of gels can be formed in the aqueous phase depending on the type and amounts of biopolymers used, and the gelation mechanism employed, including co-gelling, phase-separated, and interpenetrating systems [41]. Each of these systems provides different mechanical and functional properties, which may be useful for specific applications. The emulsifying and gelling behavior of proteins and polysaccharides can also be modulated by forming covalent conjugates or non-covalent complexes [54].

The nature of the electrostatic interactions between charged protein and polysaccharide molecules is particularly important to the formation and properties of composite emulsion gels [55]. When the proteins and polysaccharides carry opposite charges, electrostatic complexation may occur. Conversely, when they have similar charges electrostatic repulsion may occur [41,56]. Therefore, the properties of PBEGs can be modulated by tailoring protein–polysaccharide electrostatic interactions. For instance, adjusting the pH can induce opposite charges on proteins, promoting the formation of soluble electrostatic complexes with polysaccharides [20]. The choice of anionic or cationic polysaccharides used will depends on the protein’s charge characteristics under the pH conditions employed [57]. Certain kinds of enzymes can also be used to promote the gelation of protein–polysaccharide systems, resulting in gels with greater stability than those formed by proteins alone [19].

Notably, nearly all naturally occurring ionic plant-based polysaccharides are negatively charged. Therefore, to promote electrostatic attraction with plant proteins and enhance the stability of emulsion gels, gelation should be performed at a pH below the isoelectric point of the selected plant protein to ensure it is positively charged.

## 3. Characterization Methods

**Figure 2:**
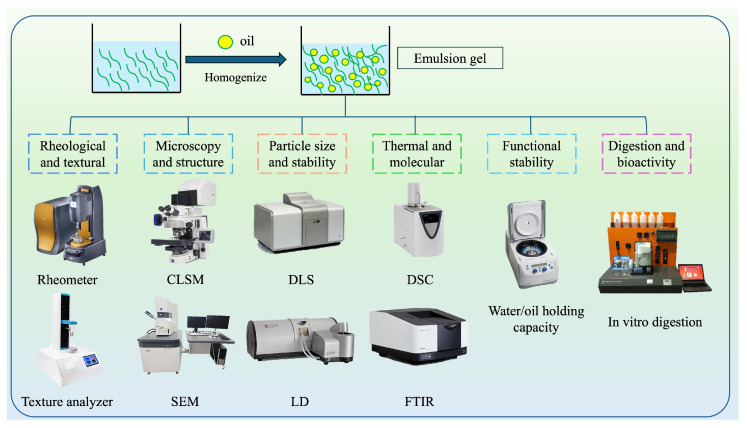
Schematic representation of the different analytical techniques used to characterize the properties of PBEGs. CLSM: confocal laser scanning microscopy; DLS: dynamic light scattering; DSC: differential scanning calorimetry; SEM: scanning electron microscopy; LD: laser diffraction; FTIR: Fourier-transform infrared spectroscopy.

Various analytical methods have been used to characterize the structural, rheological, thermal, and functional properties of emulsions (Figure 2), which are essential for understanding their formulation, behavior, and functionality [46]. Table 2 summarizes some of the most common analytical techniques used for characterizing the properties of edible PBEGs.

### 3.1. Dynamic Shear Rheology

Dynamic shear rheology is a powerful analytical tool that is commonly used to characterize the mechanical properties of emulsion gels. The tests used can be classified as smal-amplitude oscillatory shear (SAOS) or large amplitude oscillatory shear (LAOS) depending on the magnitude of the applied stress (or strain). For SAOS, the deformation of the sample is so small that the material properties are not altered, and the measured elastic properties are not strongly dependent on the magnitude of the applied stress. In contrast, for LAOS, the sample undergoes yielding or rupture, and the measured elastic properties, therefore, depend on the magnitude of the applied stress. In contrast, for LAOS, the sample undergoes yielding or rupture, and the measured elastic properties, therefore, depend on the magnitude of the applied stress. SAOS and LAOS tests are typically used to measure the storage modulus (G′) and loss modulus (G″) of a sample, which represent the elastic and viscous components of the modulus, respectively. The ratio of the viscous-to-elastic components, known as tan (δ) (=G″/G′), provides a measure of the relative importance of these two components, as follows: tan (δ) < 1 indicates a gel with elastic-dominant behavior, whereas tan (δ) > 1 indicates a gel with viscous-dominant behavior. In addition, strain sweep and strain frequency tests provide insights into the linear viscoelastic region, structural breakdown, and dynamics of gels under stress [39]. Measurements can also be carried out as a function of temperature by heating and/or cooling the sample under controlled conditions, which is useful for characterizing the behavior of heat- or cold-set gels [65]. Dynamic rheological studies have shown that biopolymer properties (such as type, concentration, and interactions), droplet properties (such as concentration and interfacial properties), solution properties (such as pH and ionic strength), and crosslinking conditions (such as temperature, time, crosslinker type, and crosslinker concentration) all influence the dynamic shear rheology of emulsion gels [66]. As an example, Li et al. [65] reported that adding β-glucan to potato-protein gels altered their rheological properties, which was attributed to the impact of this polysaccharide on protein–protein interactions. In another study, dynamic shear rheology was used to investigate the impact of different kinds of polysaccharides on the properties of β-carotene-fortified whey protein-based emulsion gels [67]. This study showed that the rheological properties of the emulsion gels could be tailored by varying the type and amount of polysaccharide added.

### 3.2. Texture Analysis: Compression/Tensile Testing

Texture analysis is widely used to simulate the mechanical deformation of emulsion gels during mastication [68]. Typically, an emulsion gel with well-defined dimensions (height and cross-sectional area) is compressed and decompressed twice, and the change in force with time is recorded. Parameters such as the hardness, cohesiveness, adhesiveness, springiness, gumminess, and chewiness of the emulsion gel can then be calculated from the resulting force-time profile, which reflect the textural and sensory attributes of the gels [69]. For some products, these instrumental measurements have been related to sensory attributes determined by consumers [70]. Numerous studies have shown that the composition, structure, and processing of emulsion gels impact their textural attributes. For instance, Kothuri et al. [71] used texture analysis to investigate the cohesion and hardness of various types of emulsion gels.

### 3.3. Microscopy and Microstructure Analysis

Understanding the microstructure of PBEGs is essential for optimizing their functional properties, as the microstructure, ultimately, determines their physicochemical and functional properties [72]. The microstructure reflects the spatial distribution and interactions among oil droplets, biopolymers (proteins and polysaccharides), water, and other components within the emulsion-gel matrix.

One of the most widely used tools for visualizing the internal structure of emulsion gels is confocal laser scanning microscopy (CLSM) with fluorescence staining [73]. This technique allows for imaging of the relative locations of oils, proteins, and polysaccharides in emulsion gels by using appropriate fluorescent dyes. For instance, oil droplets are often labeled with lipophilic dyes such as Nile Red, while proteins or polysaccharides are stained with hydrophilic dyes like FITC or Rhodamine B. Typically, it is possible to obtain submicron resolution (>500 nm) using this type of optical microscopy. CLSM can be used to assess droplet size, particle size distribution, aggregation state, and gel network continuity [74]. For example, D’Alessio et al. [75] used CLSM to characterize the microstructure of emulsion gels prepared with pea protein and observed oil droplets dispersed throughout a heterogeneous protein matrix that contained many void areas, indicating the presence of discontinuities within the hydrogel matrix in the aqueous phase. Similarly, Baune et al. [76] used CLSM to show that oil droplets were distributed throughout a soy-protein matrix in the aqueous phase of emulsion gels.

One of the main limitations of CLSM is that it cannot observe very small structural features (d < 500 nm). To provide this kind of detail, electron microscopy methods are, therefore, used [77]. Scanning electron microscopy (SEM) and cryogenic scanning electron microscopy (Cryo-SEM) have been used to provide high-resolution images of the surface morphology and internal network structure of emulsion gels [78]. Traditional SEM requires extensive sample dehydration, which may lead to considerable structural distortion of the original network structure in an emulsion gel, leading to erroneous results. In contrast, Cryo-SEM uses rapid freezing and fracturing techniques to better preserve the original microstructure of the hydrated gels [79]. These techniques are particularly useful for visualizing pore structures, polymer networks, and the spatial distribution of oil droplets within the continuous phase. In plant-based systems, Cryo-SEM has been employed to compare the structural effects of different gelation methods, such as heat-induced versus enzyme-induced gelation, on the microstructure of composite gels made from pea protein and alginate [9]. Even higher spatial resolution can be obtained using transmission electron microscopy (TEM), which involves passing an electron beam through a thin slice of sample, rather than reflecting them off the surface of a sample [80]. As an example, Yang et al. [81] has used TEM to image the microstructure of emulsion gels formed using alginate and konjac glucomannan as a gelling agent in the aqueous continuous phase. This study showed that the oil droplets were dispersed through a heterogeneous biopolymer network.

### 3.4. Particle Size

The size of the individual oil droplets, as well as their aggregation state, impact the formation, stability, and properties of emulsion gels, including their rheological behavior, appearance, creaming stability, and digestion properties [82]. Moreover, there may also be other kinds of colloidal particles in emulsion gels, whose size can impact their properties, such as protein and/or polysaccharide aggregates. Consequently, it is important to have appropriate analytical techniques to measure the particle size of emulsions and emulsion gels. These colloidal systems may contain particles that vary in dimensions from a few nanometers to hundreds of micrometers, and so it is important to select the most appropriate analytical instrument and measurement protocol to determine their particle size characteristics. The most common types of instruments used for this purpose are based on light scattering.

#### 3.4.1. Dynamic Light Scattering (DLS)

For emulsion gels with submicron-sized oil droplets—such as nanoemulsions or fine emulsions—dynamic light scattering (DLS) is the preferred technique. DLS offers high sensitivity and rapid measurement, making it suitable for monodisperse systems with narrow particle size distributions. However, in polydisperse or gelled systems, where droplet mobility is restricted and multiple scattering may occur, DLS measurements can be distorted [83].

#### 3.4.2. Laser Diffraction (LD)

For emulsion gels with droplet sizes in the range of 1–100 μm—the most common size range in food systems—laser diffraction (LD) is the most widely used technique. LD determines volume-based droplet size distributions by analyzing the intensity and angle of laser light scattered by the droplets. It is well-suited for polydisperse systems, offering a broad detection range (0.1–2000 μm) and can be combined with wet dispersion units to minimize agglomeration [84]. In practice, LD has been applied to characterize the droplet size of emulsion gels formed with natural polysaccharides, with typical measurements falling within the 1–100 μm range [85].

#### 3.4.3. Microscopy and Image Analysis

Information about the particle size distribution and aggregation state of emulsions and emulsion gels can also be assessed using microscopy methods, such as the optical or electron microscopy methods discussed in Section 3.3. After a microscopy image is obtained, then it can be analyzed using appropriate image analysis software (such as ImageJ (https://imagej.net/ij/, accessed on 22 July 2025) from the National Institutes of Health, USA), which can provide quantitative information about particle size, shape, concentration, and spatial organization. This approach is particularly useful for studying the large, irregular-shaped particles found in some PBEGs during their preparation [86].

### 3.5. Zeta-Potential Analysis

The zeta potential, which reflects the electrical potential at the shear plane of particles, is a key indicator of the stability and interactions of the particles in colloidal dispersions [87]. In PBEGs, the surface charge of the oil droplets is mainly determined by the nature of the emulsifier used to form and stabilize the emulsions, as well as pH, ionic strength, and ingredient interactions [88]. For instance, the zeta potential of protein-coated oil droplets goes from strongly negative to strongly positive as the pH is reduced from above to below their isoelectric point [89]. The zeta potential of colloidal particles is commonly measured using electrophoretic light scattering (ELS). Generally, a higher absolute zeta potential (|ζ| > 30 mV) indicates stronger electrostatic repulsion between particles and improved resistance to aggregation [90]. The magnitude and sign of the droplet charge also impacts their interactions with the surrounding biopolymer matrix in the aqueous phase [91]. In some cases, changes in zeta potential are used to promote emulsion gel formation or to alter their properties. For example, Limpisophon et al. [92] reported that the gel strength of mung bean protein-stabilized emulsion gels could be manipulated by adjusting the zeta potential of the protein-coated oil droplets.

### 3.6. Thermal Analysis

The thermal behavior of PBEGs is essential for predicting their processing performance and storage stability [93]. The most common analytical technique used to investigate the thermal transitions of emulsion gels is differential scanning calorimetry (DSC). However, dynamic shear rheology with controlled temperature sweeping is often used for this purpose too (Section 3.1).

DSC is typically used to measured endothermic and/or exothermic transitions in food systems when they are heated or cooled under controlled conditions [94]. For emulsion gels, these transitions include denaturation, gelatinization, helix-coil transitions, melting, and crystallization phenomena [95]. As an example, DSC has been used to measure the denaturation temperatures (T_d_) of soy or pea proteins used to formulate PBEGs, as well as the gelation temperatures and enthalpy changes (ΔH) of polysaccharides, like agar and carrageenan [96]. In another study, Zhang et al. [97] used DSC to study the freeze–thaw stability of emulsion gels containing soy protein and maltose. Similarly, Luo et al. [98] DSC to study starch gelatinization in emulsion gels during heating.

### 3.7. Characterization of Functional Groups and Interactions

Fourier-transform infrared spectroscopy (FTIR) and Raman spectroscopy have been used to evaluate the molecular composition and interactions of emulsion gels [99]. The techniques are based on the fact that specific peaks in infrared or Raman spectra correspond to specific functional groups, and changes in the height and position of the peaks provides information about their interactions with their environment. FTIR can also be used to provide information about the secondary structure of proteins, such as the relative amounts of α-helix, β-sheet, β-turn, and random coil regions, using suitable analysis of the spectra obtained. This method is mainly based on the measurement of absorption bands that correspond to protein structures, such as the amide I (~1650 cm^−1^) and amide II (~1540 cm^−1^) regions on the spectra [100]. Insights into the nature of the molecular interactions in emulsion gels, such as hydrogen bonding, electrostatic forces, or hydrophobic interactions, can often be obtained by measuring changes in the position or intensity of characteristic peaks, indicating interfacial interactions [101]. For example, Liang et al. [102] reported that the addition of sodium alginate altered the amide region in protein-based emulsion gels, suggesting a conformational rearrangement of the proteins that contributed to enhanced gel stability.

Raman spectroscopy serves as a complementary technique to FTIR and allows for non-destructive investigation of molecular vibrations and structural transitions in hydrated systems [103]. It is particularly useful for analyzing disulfide bond formation, the microenvironment of aromatic amino acids, and polysaccharide conformational changes [104]. Compared to FTIR, Raman spectroscopy is less sensitive to water interference, making it better suited for studying high-moisture emulsion gels.

### 3.8. Water- and Oil-Holding Capacities (WHC and OHC)

The ability of emulsion gels to retain fluids is important for many of their applications, as it affects their rheology, stability, appearance, and sensory attributes. The two most important fluids in emulsion gels are usually water and oil, which can be characterized by the water-holding capacity (WHC) and oil-holding capacity (OHC) [105]. These properties are primarily governed by the internal structure of the gel, including network porosity, interfacial composition, and interactions among biopolymers [106]. Accurate characterization of WHC and OHC values is, therefore, essential for optimizing the formulation and functional performance of PBEGs.

WHC refers to the gel’s ability to retain water when subjected to external forces such as centrifugation or gravity. A stronger and denser gel network typically exhibits superior water retention [107]. Zhao et al. [108] reported that the incorporation of konjac glucomannan into soy-protein-based gels significantly improved their WHC, which was attributed to the effective entrapment of water within the pores in the fine gel matrix.

OHC refers to the ability of the gel matrix to trap and retain oil droplets, thereby preventing oil leakage during storage and processing [109]. In some applications, a high OHC is essential to maintain emulsion stability and minimize oil phase separation. Some of the main factors influencing the OHC of emulsion gels include the interactions between the emulsifier-coated oil droplets and surrounding hydrogel matrix, the strength of the emulsifier coating around the oil droplets, and the gel network’s ability to trap oil droplets [110]. Liu et al. [111] reported that emulsion gels formed by pea protein and low methoxyl pectin exhibited good OHC, which was attributed to electrostatic complexation that enhanced the structural integrity of the gel matrix surrounding the oil droplets.

### 3.9. In Vitro Digestion Studies

PBEGs have been widely explored for their potential application as delivery systems for bioactive compounds, such as vitamins, nutraceuticals, or omega-3 fatty acids [112]. Consequently, a good understanding of their structural evolution and release behavior during digestion within the human gastrointestinal tract is required to create emulsion-gel-based delivery systems with good bioavailability [111]. Currently, static and dynamic in vitro digestion models (e.g., the INFOGEST static model) are commonly employed to provide insights into the gastrointestinal fate of emulsion gels. These digestion models are typically used in combination with physicochemical analyses, biochemical assays, and microstructural observations, to comprehensively evaluate the digestive behavior of emulsion gels [113].

Simulated gastrointestinal digestion models are commonly designed to replicate the oral, gastric, and intestinal phases of the human gut by incorporating appropriate digestive enzymes (e.g., amylase, pepsin, trypsin, and lipase), minerals, bile salts, and pH conditions. Moreover, the incubation periods, mechanical forces, and temperature in each gastrointestinal region are simulated. These in vitro models enable the investigation of gel degradation, lipid digestion kinetics, and protein digestion kinds, and bioactive release and solubilization [114]. For instance, Nie et al. [115] replaced some of the meat in sausages with a pea-protein-based emulsion gel and then studied their in vitro gastrointestinal behavior. They found that the plant-based gels broke down during passage through the simulated gastrointestinal tract, and that the concentration of soluble proteins in the gastrointestinal fluids increased. Similarly, Cui et al. [116] used camellia seed proteins to formulate EGCG-loaded emulsion gels. The in vitro digestion results showed that encapsulation in the emulsion gel significantly delayed EGCG release, reducing it from around 81% to 27%. Moreover, the encapsulated EGCG had a 31% higher bioaccessibility than free EGCG.

### 3.10. Sensory Properties

Sensory characteristics are critical for consumer acceptance of PBEGs, especially when used as fat replacers in food products. Traditional sensory evaluations rely on trained expert panels or consumer testing to qualitatively and quantitatively assess these attributes [117]. For example, Badar et al. [118] developed a PBEG using walnut oil, peanut oil, and chia powder to replace buffalo back fat in hamburger formulations. Sensory evaluation by a trained panel indicated that appearance, aroma, flavor, and juiciness were all within an acceptable range. Instrumental techniques—such as tribology, texture profile analysis, and rheology—are increasingly being integrated with sensory testing to establish correlations between perceived sensations and physicochemical properties. Li et al. [119] formulated an emulsion gel stabilized by pomelo fiber and soy protein isolate as a cream substitute for ice cream production. The product was reported to have favorable attributes in terms of texture, freshness, and sweetness, which were supported by TPA measurements of hardness, chewiness, and stickiness. These examples highlight the value of combining sensory and instrumental approaches to develop predictive models of consumer perception.

## 4. Application of PBEGs in Food

**Figure 3:**
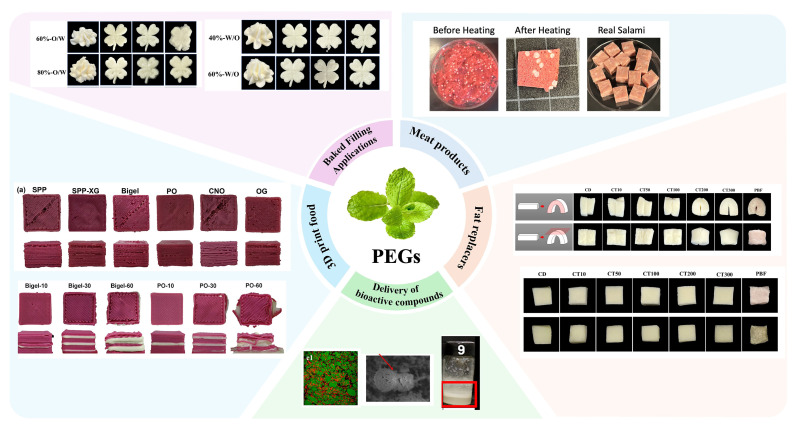
Application of PBEGs in different kinds of foods. Fat replacers adapted from Ref. [63], with permission from Elsevier. Meat products adapted from Ref. [120], with permission from Elsevier. Delivery of bioactive compounds adapted from Ref. [121], with permission from Elsevier. 3D printed foods adapted from Ref. [122], with permission from Elsevier. Baked filling applications adapted from Ref. [123], with permission from Elsevier.

A wide range of potential applications of PBEGs in the food industry have already been explored (Figure 3). In this section, we provide a brief overview of some of the most common ones to highlight their potential in foods.

### 4.1. Fat Replacers in Low-Fat Foods

To meet the growing demand for healthier food products, the food industry is actively exploring fat replacement strategies aimed at reducing total and saturated fat contents, while still maintaining desirable functional and sensory qualities [44]. Among these strategies, PBEGs have emerged as promising fat replacers due to their ability to mimic the creaminess and texture of conventional fats, along with advantages such as lower caloric density, a more favorable fatty acid profile, better sustainability, and lower environmental impact [117]. These systems are typically composed of lipid droplets embedded with a hydrogel matrix formed from plant-derived proteins and/or polysaccharides, which may be crosslinked using a variety of approaches, including thermal, ionic, or enzymatic gelation [118]. By trapping the oil droplets within a three-dimensional gel network, PBEGs can deliver fat-like properties such as cohesiveness and thermal stability.

Limpisophon et al. [92] developed plant-based emulsion gels using mung bean proteins that exhibited mouthfeel properties comparable to those of animal fat. Modification of the mung bean hydrogels by incorporating 0.3% sodium carbonate followed by heat treatment reduced protein aggregation and improved the permeability and stability of these emulsion gels. Christoph et al. [62] prepared high-internal-phase emulsions (HIPEs) using soy proteins and sunflower oil and demonstrated that crosslinking of oil droplets via hydrogen bonding (using tannic acid) or electrostatic interactions (using calcium ions) formed a secondary network that mimicked both the small- and large-deformation rheological behavior of bovine adipose tissue. Choi et al. [63] created a thermally-induced emulsion gel based on soy oil and soy protein, using rennet and transglutaminase to achieve protein crosslinking. The resulting gel successfully simulated the thermal profile and elastic texture of pork fat. Optimized protein crosslinking improved the elasticity of the plant-based fat analog, eliminating the brittleness typically associated with such systems and enhancing their similarity to animal fat.

### 4.2. Plant-Based Meat Products

With growing consumer demand for sustainable, ethical, and health-conscious food alternatives, the development of plant-based meat analogs is rapidly advancing [64]. One of the major technical challenges lies in replicating the complex texture, juiciness, and fat distribution of real meat [124]. PBEGs, composed of oil droplets embedded within semi-solid plant protein/polysaccharide matrices, have emerged as promising candidates for mimicking the functional roles of many meat products [125]. In these systems, the oil droplets are used to mimic the adipose tissue, whereas the biopolymers are used to mimic the lean tissue.

PBEGs have been engineered to simulate intramuscular fat marbling in meat, offering desirable lubrication, mouthfeel, and flavor release characteristics during cooking and mastication. For instance, Hu et al. [120] developed an emulsion gel based on potato protein and a cold-set polysaccharide gel, which successfully replicated the lean meat regions in salami-type sausages. Increasing the protein content enhanced the mechanical strength of the lean meat analogs. Unlike liquid oils, PBEGs retain a semi-solid state at room or refrigerated temperatures, helping maintain their structural integrity like real meat.

The incorporation of PBEGs into plant-based meat products can effectively enhance their structural integrity, reduce cooking loss, and improve water and oil retention [126]. For instance, Penchalaraju et al. [127] developed fried plant-based meatballs using cowpea-protein-based emulsion gels, which showed similar oil uptake, protein solubility, texture, flavor, and color as conventional meatballs. These effects were attributed to the encapsulation of oil droplets within the gel network, allowing for controlled lipid release and uniform distribution. Czapalay et al. [128] prepared PBEGs using pea starch and chickpea flour, which exhibited thermal properties in the range of 5–85 °C similar to animal meat, replicating key functional attributes of beef tissue.

One of the main advantages of PBEGs is their excellent tunability. Their hardness, melting behavior, and viscoelasticity can be controlled by adjusting the protein/polysaccharide type and amount, oil droplet size and concentration, emulsifier type, and gelling method (e.g., ionic, thermal, or enzymatic). This adaptability enables their application across a wide range of plant-based meat products, including burgers, sausages, and nuggets, as well as possibly whole cut analogs like chicken breast, beef steak, fish fillets, or pork chops. Tahir et al. [129] used three different crosslinking enzymes to modify the properties of emulsion gels formed using pea protein as a gelling agent. By controlling the crosslinking conditions, the physicochemical properties of the PBEGs could be made to be somewhat similar to those of conventional meat. In another study, modulation of pea proteins through pH, heat, and sonication treatments has been found to enhance the mechanical strength and overall quality of plant-based emulsion gels [130].

### 4.3. Delivery of Bioactive Compounds

PBEGs have garnered increasing attention in the food field as delivery systems for bioactive compounds such as vitamins, nutraceuticals, omega-3 fatty acids, and probiotics [131]. These structured emulsions are typically formed by trapping oil droplets within a gel matrix containing plant proteins and/or polysaccharides. Typically, hydrophobic bioactives are trapped inside the oil droplets, whereas hydrophilic ones are trapped in the surrounding gel matrix. The composition and structure of the emulsion gels can be optimized to improve the stability, release profile, and bioaccessibility of the encapsulated bioactive agents [132].

Many bioactive compounds are prone to chemical degradation under environmental stressors, such as heat, light, and oxygen. The gel matrix in PBEGs can be designed to protect these compounds from external factors, thereby enhancing their chemical stability [133]. For instance, Yan et al. [121] developed a dual-crosslinked emulsion gel using zein and alginate as gelling agents to encapsulate curcumin and resveratrol. Compared to the single-crosslinked systems, the dual-crosslinked system significantly improved the photostability of both polyphenols. Similarly, Muñoz-González et al. [134] constructed a plant-based emulsion gel for delivering phenolic extracts from grape seed and olive, achieving high thermal stability and maintaining metabolite integrity. Zhou et al. [135] designed a dual plant-based emulsion gel for incorporating probiotics, which not only enhanced emulsion stability but also improved probiotic survival during intestinal transit.

The release properties of encapsulated bioactive agents can also be controlled by altering the composition and structure of PBEGs. For instance, Farooq et al. [136] reported that emulsion gels containing catechins and camellia oil bodies delayed the release of these bioactive substances under simulated gastric conditions due to the presence of a protein network in the aqueous phase. As a result, the free fatty acid release was delayed in the small intestine, but the final bioaccessibility of the hydrophobic bioactives increased. Similarly, Jiang et al. [137] developed a cold-set emulsion gel using soy protein to encapsulate vitamin D_3_, which significantly enhanced its release during the intestinal phase, achieving a release extent of 55.5 ± 1.6%. Other researchers reported that encapsulating vitamin D_3_ in plant-based emulsion gels (PBEGs) improved its bioaccessibility and bioavailability, with the AUC_0–24h_ value increasing by over 30% [138]. Abu-El Khair et al. [139] incorporated probiotics into PBEGs and found that encapsulation delayed the oxidative degradation of wheat germ oil and enhanced probiotic viability, indicating the great potential of PBEGs in products such as yogurt.

### 4.4. Additive Manufacturing: 3D Printed Foods

3D food printing (also known as additive manufacturing) is an emerging technology that can be used for designing foods with customizable compositions, colors, shapes, sizes, textures, and nutritional profiles. However, it is critical to have suitable edible inks to formulate these products. PBEGs are particularly suited for extrusion-based 3D printing applications due to their favorable rheological attributes, as well as the ability to encapsulate a wide range of ingredients, including colors, flavors, preservatives, nutrients, and nutraceuticals [105,106].

A key requirement for successful 3D printing is that the printing material exhibits appropriate rheological behavior, such as shear-thinning and a sufficient yield stress [140]. This is so that the edible inks can be extruded through the printing nozzle and then rapidly set on the printing platform and retain their shape. PBEGs can be formulated to meet these criteria by altering their composition, structure, or gelling mechanism. For example, Guo et al. [122] demonstrated that emulsion gels based on spirulina protein nanoparticles could be 3D printed through a single-nozzle system, resulting in novel plant-based meat products with high printing fidelity and low hardness for improved chewability. In another study, Mohammadi et al. [141] grafted phenolic compounds onto soy proteins to improve their performance in creating emulsion-gel-based edible inks with properties appropriate for 3D food printing applications.

Despite the promising potential of PBEGs in 3D printing, several challenges remain, including poor adhesion, post-printing shrinkage and deformation, and limited compatibility with industrial-scale applications [142]. Future research is, therefore, needed to improve their printing performance.

### 4.5. Baked Filling Applications

PBEGs are also being explored as alternatives to high-fat fillings in baked goods, such as butter, cream, and toppings, with the aim of improving their healthiness [143]. The application of PBEGs in baked products relies primarily on them having good thermal stability, appropriate mechanical properties, and the ability to retain moisture and lipids at elevated temperatures while also delivering a desirable mouthfeel and flavor release. For example, Mefleh et al. [144] developed PBEGs composed of pea proteins and extra virgin olive oil to replace cream cheese. They were able to mask the undesirable legume flavors associated with the pea proteins by incorporating spices. The final product had a spreadability index comparable to cream cheese but with a healthier fat profile. Similarly, Zhang et al. [145] formulated a PBEG to replace butter in baked goods. They showed that its solid-like properties could compensate for butter’s role in aerating and stabilizing bread crumb structure, thereby preserving desirable baking performance. Moreover, the PBEG-based butter alternatives enhanced the bioaccessibility of the fatty acids in the baked goods [146]. However, more studies are still required in this area using real baked goods and carrying out detailed performance and sensory studies.

## 5. Future Perspectives

Despite significant advancements in the formulation, characterization, and applications of plant-based emulsion gels (PBEGs), their widespread adoption and industrialization still face several challenges (Table 3). Future research may focus on addressing the following areas:

(1) An improved understanding of the factors impacting the formation of different kinds of PBEGs is still needed, particularly the nature of the interactions between lipid droplets, polysaccharides, and proteins. Advanced techniques, such as small-angle X-ray scattering (SAXS), nuclear magnetic resonance (NMR), and molecular dynamics (MD) simulations, may lead to appreciable advances in this area. For instance, NMR relaxation studies provide information about the mobility of water and oil molecules in emulsion gels, which can provide insights into their structure and properties [122]. MD simulations can model the molecular interactions of proteins, polysaccharides, lipids, and water, providing insights into the molecular basis of emulsion gel properties [147].
(2) Greater attention should be given to exploring how natural bioactive compounds (e.g., vitamins, minerals, nutraceuticals, ω-3 fatty acids, and probiotics) affect the physicochemical properties and nutritional profiles of PBEGs, with the aim of striking a balance between nutritional value and desirable sensory attributes.
(3) Poor sensory attributes remain a major barrier to industrial application. This could be addressed by employing advanced flavor enhancement strategies, such as reducing oral friction and incorporating optimized flavoring agents.

## 6. Conclusions

Plant-based emulsion gels align well with the growing demand for more sustainable and healthy food ingredients. They can be used to formulate analogs of animal products (like meat, fish, eggs, and adipose tissue), as fat replacers, delivery systems for bioactive agents, and edible inks for 3D food printing. This review summarizes recent advances in the preparation, characterization, and applications of PBEGs in food systems. The information presented may support the rational design and application of innovative plant-based food products. However, several challenges still remain, including the need for a more rational understanding of PBEG formation and properties, as well as the need for better integration of active ingredients to enhance sensory and nutritional quality. Addressing these issues will require interdisciplinary collaboration across food chemistry, materials science, nutrition, food engineering, sensory science, and consumer studies. In summary, PBEGs have great potential for the design of a broad range of healthier and more sustainable food products.

## Figures and Tables

**Table 1:** Examples of studies of emulsion gels in foods. The gels are typically prepared using different gelling agents and preparation methods.

Source	Gelling Agent	Preparation Method	Main Interactions	Ref.
Protein-based	Pea protein	Heat (95 °C)	Disulfide bonds	[9]
	Soy protein	Calcium ions	Ionic bonds Hydrophobic interactions	[10]
	Whey protein	Acidify	Hydrogen bonds Hydrophobic interactions	[11]
	Pea protein	Transglutaminase pH 7, room temperature	Covalent bonds	[12]
	Hempseed protein	Genipin Room temperature for 4 h	Covalent bonds	[13]
Polysaccharide-based	Agar	Heat (60 °C)	Physical crosslinked network	[14]
	Chitosan	Alginate and calcium ions Room temperature	Ionic bond	[15]
	*Flammulina velutipes* polysaccharide	Adjust pH to 9 90 °C for 30 min	Hydrogen bond interactions Hydrophobic interactions	[16]
	Corn fiber gum	Peroxidase Room temperature		[17]
Protein–polysaccharide-based	Carrageenan Gum arabic–soybean protein	Hold at 90 °C for 30 min Add calcium ions	Ionic bonds Disulfide bonds Hydrogen bonds Hydrophobic interactions	[18]
	Soy protein and polysaccharide microgels	Add transglutaminase 37 °C for 60 min	Covalent bonds Hydrophobic interactions Ionic bonds Hydrogen bonds	[19]
	*Artemisia sphaerocephala* Krasch polysaccharides Whey protein fibrils	Add iron ions and place at room temperature for 72 h	Electrostatic interactions	[20]
	Flaxseed protein Chitosan	Add calcium ions, low acid, and low-temperature, 40 °C gel	Electrostatic interactions	[21]

**Table 2:** Characterization techniques used for emulsion gels in food.

Category	Method	Measured Parameter	Advantages	Limitations	Ref.
Rheological and Textural	Oscillatory rheology	G′ and G′′	Sensitive to gel strength and viscoelasticity	Requires precise temperature and shear control	[58]
	Texture Profile Analysis (TPA)	Hardness, cohesiveness, springiness	Mimics consumer texture perception	May not detect subtle network differences	[59]
Microscopy and Structure	CLSM (Confocal Laser Scanning Microscopy)	Droplet distribution, gel microstructure	3D visualization with dye labeling	Requires fluorescence labeling; limited penetration depth	[60]
	SEM/Cryo-SEM	Network morphology, surface features	High-resolution imaging	Sample dehydration or freezing may alter ative structure	[53]
Particle Size	Dynamic Light Scattering (DLS)	Droplet size distribution (<1 µm)	Rapid and sensitive	Limited accuracy for polydisperse or gelled systems	[54]
	Laser Diffraction Particle Sizer (LD)	Droplet size distribution (1–100 µm)	Fast and efficient	Not suitable for complete 3D gel networks	[61]
Zeta Potential	Zetasizer	Surface charge, colloidal stability	Predicting flocculation or aggregation	Sensitive to ionic strength and pH	[62]
Thermal and Molecular	DSC (Differential Scanning Calorimetry)	Protein denaturation, gel–sol transitions	Quantitative thermal behavior analysis	Cannot identify specific molecular interactions	[25]
	FTIR/Raman Spectroscopy	Hydrogen bonding, protein–polysaccharide interaction	Identifies specific functional group changes	Requires interpretation of overlapping peaks	[63]
Functional Stability	Water/Oil-Holding Capacity	WHC, OHC via centrifugation or filtration	Simple, directly relevant to food applications	May not distinguish mechanisms of phase retention	[64]

**Table 3:** Important challenges of PBEGs in food.

Field of Research	Key Challenges
Structure formation mechanism	Limited analytical tools and insufficient understanding of lipid–polysaccharide–protein–water interactions.
Functional ingredient integration	Limited understanding of the impact of bioactive compound type (e.g., vitamins, nutraceuticals, prebiotics, and probiotics) on emulsion gel structure, stability, and properties.
Sensory quality	Limited understanding of the factors impacting the appearance, texture, aroma, taste, and mouthfeel of emulsion gels. Limited knowledge of how to design emulsion gels for specific food applications.

## Data Availability

No new data were generated in this review article.

## References

[1] Li P., Guo C., Li X., Yuan K., Yang X., Guo Y., Yang X. (2021). Preparation and Structural Characteristics of Composite Alginate/Casein Emulsion Gels: A Microscopy and Rheology Study. Food Hydrocoll..

[2] Wang L., Zhang H., Li H., Zhang H., Chi Y., Xia N., Li Z., Jiang L., Zhang X., Rayan A.M. (2022). Fabrication and Digestive Characteristics of High Internal Phase Pickering Emulsions Stabilized by Ovalbumin-Pectin Complexes for Improving the Stability and Bioaccessibility of Curcumin. Food Chem..

[3] Li X., Cao Q., Liu G. (2025). Advances, Applications, Challenges and Prospects of Alternative Proteins. J. Food Compos. Anal..

[4] Abe-Inge V., Aidoo R., Moncada De La Fuente M., Kwofie E.M. (2024). Plant-Based Dietary Shift: Current Trends, Barriers, and Carriers. Trends Food Sci. Technol..

[5] Zhi L., Liu Z., Wu C., Ma X., Hu H., Liu H., Adhikari B., Wang Q., Shi A. (2023). Advances in Preparation and Application of Food-Grade Emulsion Gels. Food Chem..

[6] Cen S., Meng Z. (2024). Advances of Plant-Based Fat Analogs in 3D Printing: Manufacturing Strategies, Printabilities, and Food Applications. Food Res. Int..

[7] Li R., Guo Y., Dong A., Yang X. (2023). Protein-Based Emulsion Gels as Materials for Delivery of Bioactive Substances: Formation, Structures, Applications and Challenges. Food Hydrocoll..

[8] Cen S., Li S., Meng Z. (2024). Advances of Protein-Based Emulsion Gels as Fat Analogues: Systematic Classification, Formation Mechanism, and Food Application. Food Res. Int..

[9] Hoehnel A., Zannini E., Arendt E.K. (2022). Targeted Formulation of Plant-Based Protein-Foods: Supporting the Food System’s Transformation in the Context of Human Health, Environmental Sustainability and Consumer Trends. Trends Food Sci. Technol..

[10] Harper B.A., Barbut S., Lim L.-T., Marcone M.F. (2013). Characterization of ‘Wet’ Alginate and Composite Films Containing Gelatin, Whey or Soy Protein. Food Res. Int..

[11] Cui H., Mu Z., Xu H., Bilawal A., Jiang Z., Hou J. (2024). Seven Sour Substances Enhancing Characteristics and Stability of Whey Protein Isolate Emulsion and Its Heat-Induced Emulsion Gel under the Non-Acid Condition. Food Res. Int..

[12] Zhan F., Tang X., Sobhy R., Li B., Chen Y. (2022). Structural and Rheology Properties of Pea Protein Isolate-stabilised Emulsion Gel: Effect of Crosslinking with Transglutaminase. Int. J. Food Sci. Tech..

[13] Wang Q., Jiang J., Xiong Y.L. (2019). Genipin-Aided Protein Cross-Linking to Modify Structural and Rheological Properties of Emulsion-Filled Hempseed Protein Hydrogels. J. Agric. Food Chem..

[14] Wang Z., Neves M.A., Kobayashi I., Uemura K., Nakajima M. (2013). Preparation, Characterization, and in Vitro Gastrointestinal Digestibility of Oil-in-Water Emulsion-Agar Gels. Biosci. Biotechnol. Biochem..

[15] Zheng B., Zhang Z., Chen F., Luo X., McClements D.J. (2017). Impact of Delivery System Type on Curcumin Stability: Comparison of Curcumin Degradation in Aqueous Solutions, Emulsions, and Hydrogel Beads. Food Hydrocoll..

[16] Wang Z., Li L., Jia F., Wu J., Jin W., Zhao W., Cao J., Cheng Y., Shi L., Yun S. (2025). Exploring the Effect of pH-Shifting on the Gel Properties and Interaction of Heat-Induced Flammulina Velutipes Polysaccharide-Porcine Myofibrillar Protein for Improving the Quality of Flammulina Velutipes-Pork Patties. Food Chem..

[17] Liu Y., Yadav M.P., Yin L. (2018). Enzymatic Catalyzed Corn Fiber Gum-Bovine Serum Albumin Conjugates: Their Interfacial Adsorption Behaviors in Oil-in-Water Emulsions. Food Hydrocoll..

[18] Gao T., Wu X., Gao Y., Teng F., Li Y. (2024). Construction of Emulsion Gel Based on the Interaction of Anionic Polysaccharide and Soy Protein Isolate: Focusing on Structural, Emulsification and Functional Properties. Food Chem. X.

[19] Wang Z., Long J., Zhang C., Hua Y., Li X. (2025). Effect of Polysaccharide on Structures and Gel Properties of Microgel Particle Reconstructed Soybean Protein Isolate/Polysaccharide Complex Emulsion Gels as Solid Fat Mimetic. Carbohydr. Polym..

[20] Ju Q., Li N., McClements D.J., Liu N., Lu L., Yao X. (2025). Emulsion Gels Formed by Complexation or Phase-Separation Using *Artemisia sphaerocephala* Krasch. Polysaccharide/Whey Protein Isolate Fibrils: Fabrication and Applications. Food Hydrocoll..

[21] Wang Y.-S., Ding M.-Y., Chen Y., Hu X.-T., Zhang Y.-X., Fang Z.-W., Chen H.-H. (2025). Double Cross-Linked Emulsion Gels Stabilized by Flaxseed Protein and Chitosan: Effects of CaCO3 Content on Gel Properties, Stability and Curcumin Digestive Characteristics. Food Chem..

[22] Liu Z., Ren X., Cheng Y., Zhao G., Zhou Y. (2021). Gelation Mechanism of Alkali Induced Heat-Set Konjac Glucomannan Gel. Trends Food Sci. Technol..

[23] Schmitt C., Silva J.V., Amagliani L., Chassenieux C., Nicolai T. (2019). Heat-Induced and Acid-Induced Gelation of Dairy/Plant Protein Dispersions and Emulsions. Curr. Opin. Food Sci..

[24] Dickinson E. (2012). Emulsion Gels: The Structuring of Soft Solids with Protein-Stabilized Oil Droplets. Food Hydrocoll..

[25] Hu X., Xiang X., Ju Q., Li S., Julian McClements D. (2024). Impact of Lipid Droplet Characteristics on the Rheology of Plant Protein Emulsion Gels: Droplet Size, Concentration, and Interfacial Properties. Food Res. Int..

[26] Teng C., Campanella O.H. (2023). A Plant-Based Animal Fat Analog Produced by an Emulsion Gel of Alginate and Pea Protein. Gels.

[27] Basak S., Singhal R.S. (2024). Inclusion of Konjac Glucomannan in Pea Protein Hydrogels Improved the Rheological and in Vitro Release Properties of the Composite Hydrogels. Int. J. Biol. Macromol..

[28] Yeo G.C., Keeley F.W., Weiss A.S. (2011). Coacervation of Tropoelastin. Adv. Colloid. Interface Sci..

[29] Wang Y., Zhou K., Ye C., Shang Y., Wang F. (2025). Preparation and Characterization of Microemulsions Formulated with PEG Fatty Acid Glycerides. Colloids Surf. B Biointerfaces.

[30] Zhao D., Sun L., Wang Y., Liu S., Cao J., Li H., Liu X. (2024). Salt Ions Improve Soybean Protein Isolate/Curdlan Complex Fat Substitutes: Effect of Molecular Interactions on Freeze-Thaw Stability. Int. J. Biol. Macromol..

[31] Nephomnyshy I., Rosen-Kligvasser J., Davidovich-Pinhas M. (2020). The Development of a Direct Approach to Formulate High Oil Content Zein-Based Emulsion Gels Using Moderate Temperatures. Food Hydrocoll..

[32] Mantovani R.A., Cavallieri Â.L.F., Cunha R.L. (2016). Gelation of Oil-in-Water Emulsions Stabilized by Whey Protein. J. Food Eng..

[33] Lin D., Kelly A.L., Miao S. (2022). The Impact of pH on Mechanical Properties, Storage Stability and Digestion of Alginate-Based and Soy Protein Isolate-Stabilized Emulsion Gel Beads with Encapsulated Lycopene. Food Chem..

[34] Masiá C., Ong L., Logan A., Stockmann R., Gambetta J., Jensen P.E., Rahimi Yazdi S., Gras S. (2024). Enhancing the Textural and Rheological Properties of Fermentation-Induced Pea Protein Emulsion Gels with Transglutaminase. Soft Matter.

[35] Qayum A., Hussain M., Li M., Li J., Shi R., Li T., Anwar A., Ahmed Z., Hou J., Jiang Z. (2021). Gelling, Microstructure and Water-Holding Properties of Alpha-Lactalbumin Emulsion Gel: Impact of Combined Ultrasound Pretreatment and Laccase Cross-Linking. Food Hydrocoll..

[36] Zhang M., Yin L., Yan W., Gao C., Jia X. (2022). Preparation and Characterization of a Novel Soy Protein Isolate-Sugar Beet Pectin Emulsion Gel and Its Application as a Multi-Phased Nutrient Carrier. Foods.

[37] Zi Y., Shi C., Kan G., Peng J., Gong H., Wang X., Zhong J. (2024). Two Types of pH-Responsive Genipin-Crosslinked Gelatin Conjugates with High Surface Hydrophobicity for Emulsion Stabilization. Food Hydrocoll..

[38] Xu D., Hui Y.-Y., Zhang W., Zhao M.-N., Gao K., Tao X.-R., Wang J.-W. (2024). Genipin-Crosslinked Hydrogels for Food and Biomedical Applications: A Scientometric Review. Int. J. Biol. Macromol..

[39] Eghbaljoo H., Sani I.K., Sani M.A., Rahati S., Mansouri E., Molaee-Aghaee E., Fatourehchi N., Kadi A., Arab A., Sarabandi K. (2022). Advances in Plant Gum Polysaccharides; Sources, Techno-Functional Properties, and Applications in the Food Industry - A Review. Int. J. Biol. Macromol..

[40] Liu X., Chao C., Yu J., Copeland L., Wang S. (2021). Mechanistic Studies of Starch Retrogradation and Its Effects on Starch Gel Properties. Food Hydrocoll..

[41] McClements D.J. (2024). Composite Hydrogels Assembled from Food-Grade Biopolymers: Fabrication, Properties, and Applications. Adv. Colloid. Interface Sci..

[42] Ryu J., McClements D.J. (2024). Impact of Heat-Set and Cold-Set Gelling Polysaccharides on Potato Protein Gelation: Gellan Gum, Agar, and Methylcellulose. Food Hydrocoll..

[43] Jiang F., Xu X., Xiao Q., Li Z., Weng H., Chen F., Xiao A. (2024). Fabrication, Structure, Characterization and Emulsion Application of Citrate Agar. Int. J. Biol. Macromol..

[44] Niu J., Li X., McClements D.J., Ji H., Jin Z., Qiu C. (2025). Biopolymer-Based Emulsion Gels as Fat Replacers: A Review of Their Design, Fabrication, and Applications. Int. J. Biol. Macromol..

[45] Ye X., Wei L., Sun L., Xu Q., Cao J., Li H., Pang Z., Liu X. (2024). Fabrication of Food Polysaccharide, Protein, and Polysaccharide-Protein Composite Gels via Calcium Ion Inducement: Gelation Mechanisms, Conditional Factors, and Applications. Int. J. Biol. Macromol..

[46] Silva D.A., Brito A.C.F., De Paula R.C.M., Feitosa J.P.A., Paula H.C.B. (2003). Effect of Mono and Divalent Salts on Gelation of Native, Na and Deacetylated Sterculia Striata and Sterculia Urens Polysaccharide Gels. Carbohydr. Polym..

[47] Li A., Gong T., Yang X., Guo Y. (2020). Interpenetrating Network Gels with Tunable Physical Properties: Glucono-δ-Lactone Induced Gelation of Mixed Alg/Gellan Sol Systems. Int. J. Biol. Macromol..

[48] Donati I., Benegas J., Paoletti S. (2021). On the Molecular Mechanism of the Calcium-Induced Gelation of Pectate. Different Steps in the Binding of Calcium Ions by Pectate. Biomacromolecules.

[49] Hu X., Jiang Q., Du L., Meng Z. (2023). Edible Polysaccharide-Based Oleogels and Novel Emulsion Gels as Fat Analogues: A Review. Carbohydr. Polym..

[50] Evageliou V. (2000). Effect of pH, Sugar Type and Thermal Annealing on High-Methoxy Pectin Gels. Carbohydr. Polym..

[51] Zhu Y., McClements D.J., Zhou W., Peng S., Zhou L., Zou L., Liu W. (2020). Influence of Ionic Strength and Thermal Pretreatment on the Freeze-Thaw Stability of Pickering Emulsion Gels. Food Chem..

[52] Lin Q., Hu Y., Qiu C., Li X., Sang S., McClements D.J., Chen L., Long J., Xu X., Wang J. (2023). Peanut Protein-Polysaccharide Hydrogels Based on Semi-Interpenetrating Networks Used for 3D/4D Printing. Food Hydrocoll..

[53] Meng W., Sun H., Mu T., Garcia-Vaquero M. (2023). Pickering Emulsions with Chitosan and Macroalgal Polyphenols Stabilized by Layer-by-Layer Electrostatic Deposition. Carbohydr. Polym..

[54] Burgos-Díaz C., Wandersleben T., Marqués A.M., Rubilar M. (2016). Multilayer Emulsions Stabilized by Vegetable Proteins and Polysaccharides. Curr. Opin. Colloid. Interface Sci..

[55] Guo W., Ding X., Zhang H., Liu Z., Han Y., Wei Q., Okoro O.V., Shavandi A., Nie L. (2024). Recent Advances of Chitosan-Based Hydrogels for Skin-Wound Dressings. Gels.

[56] Lin D., Kelly A.L., Miao S. (2020). Preparation, Structure-Property Relationships and Applications of Different Emulsion Gels: Bulk Emulsion Gels, Emulsion Gel Particles, and Fluid Emulsion Gels. Trends Food Sci. Technol..

[57] Li M., Feng L., Dai Z., Li D., Zhang Z., Zhou C., Yu D. (2025). Improvement of 3D Printing Performance of Whey Protein Isolate Emulsion Gels by Regulating Rheological Properties: Effect of Polysaccharides Incorporation. Food Bioprocess. Technol..

[58] Al-Sharify Z.T., Al-Najjar S.Z., Anumudu C.K., Hart A., Miri T., Onyeaka H. (2025). Non-Thermal Technologies in Food Processing: Implications for Food Quality and Rheology. Appl. Sci..

[59] Grau R., Pérez A.J., Hernández S., Barat J.M., Talens P., Verdú S. (2024). Data from Chewing and Swallowing Processes as a Fingerprint for Characterizing Textural Food Product Properties. Food Bioprocess. Technol..

[60] Zhu P., Chu Y., Yang J., Chen L. (2024). Thermally Reversible Emulsion Gels and High Internal Phase Emulsions Based Solely on Pea Protein for 3D Printing. Food Hydrocoll..

[61] Scholten E. (2017). Composite Foods: From Structure to Sensory Perception. Food Funct..

[62] Christoph V., Bender D., Xia W., Domig K.J., Fuhrmann P.L. (2025). Thermo-Responsive Droplet-Droplet Interactions in High Internal Phase Emulsions: A Strategy for Adipose Tissue Substitution. Food Hydrocoll..

[63] Choi M., Choi H.W., Jo M., Hahn J., Choi Y.J. (2024). High-Set Curdlan Emulsion Gel Fortified by Transglutaminase: A Promising Animal Fat Substitute with Precisely Simulated Texture and Thermal Stability of Animal Fat. Food Hydrocoll..

[64] Liu X., Yang C., Qin J., Li J., Li J., Chen J. (2023). Challenges, Process Technologies, and Potential Synthetic Biology Opportunities for Plant-Based Meat Production. LWT.

[65] Li S., Luo M., Wannasin D., Hu X., Ryu J., Ju Q., McClements D.J. (2024). Exploring the Potential of Plant-Based Emulsion Gels Enriched with β-Glucan and Potato Protein as Egg Yolk Alternatives. Food Hydrocoll..

[66] Liu X.T., Zhang H., Wang F., Luo J., Guo H.Y., Ren F.Z. (2014). Rheological and Structural Properties of Differently Acidified and Renneted Milk Gels. J. Dairy. Sci..

[67] Li M., Feng L., Xu Y., Nie M., Li D., Zhou C., Dai Z., Zhang Z., Zhang M. (2023). Rheological Property, β-Carotene Stability and 3D Printing Characteristic of Whey Protein Isolate Emulsion Gels by Adding Different Polysaccharides. Food Chem..

[68] Yang Z., He X., Song Y., Zhang W., Chen L., Jiang L., Huang Z., Tian T. (2024). Fabrication and Characterization of Novel Curcumin-Loaded Thermoreversible High Amylose Maize Starch Emulsion Gel. Int. J. Biol. Macromol..

[69] Cappa E.R., Sponton O.E., Olivares M.L., Santiago L.G., Perez A.A. (2024). Effects of pH, Kappa-Carrageenan Concentration and Storage Time on the Textural and Rheological Properties of Pea Protein Emulsion-Gels. Int. J. Food Sci. Technol..

[70] Gåmbaro A., Varela P., Giménez A., Aldrovandi A., Fiszman S.M., Hough G. (2002). TEXTURAL QUALITY OF WHITE PAN BREAD BY SENSORY AND INSTRUMENTAL MEASUREMENTS. J. Texture Stud..

[71] Kothuri V., Keum D.H., Lee H.J., Han J.H., Han S.G. (2024). Emulsion Gels with Chia Flour Enhance Quality of Plant-Based Meat Analogs by Reducing Fat and Improving Texture. LWT.

[72] Wei L., Ren Y., Huang L., Ye X., Li H., Li J., Cao J., Liu X. (2024). Quality, Thermo-Rheology, and Microstructure Characteristics of Cubic Fat Substituted Pork Patties with Composite Emulsion Gel Composed of Konjac Glucomannan and Soy Protein Isolate. Gels.

[73] Pei Z., Wang H., Xia G., Hu Y., Xue C., Lu S., Li C., Shen X. (2023). Emulsion Gel Stabilized by Tilapia Myofibrillar Protein: Application in Lipid-Enhanced Surimi Preparation. Food Chem..

[74] McClements D.J., Grossmann L. (2021). A Brief Review of the Science behind the Design of Healthy and Sustainable Plant-Based Foods. npj Sci. Food.

[75] D’Alessio G., Iervese F., Valbonetti L., Faieta M., Pittia P., Di Mattia C.D. (2024). Tailoring Pea Proteins Gelling Properties by High-Pressure Homogenization for the Formulation of a Model Spreadable Plant-Based Product. LWT.

[76] Baune M.-C., Schroeder S., Witte F., Heinz V., Bindrich U., Weiss J., Terjung N. (2021). Analysis of Protein-Network Formation of Different Vegetable Proteins during Emulsification to Produce Solid Fat Substitutes. Food Meas..

[77] Liu C., Li Y., Liang R., Sun H., Wu L., Yang C., Liu Y. (2023). Development and Characterization of Ultrastable Emulsion Gels Based on Synergistic Interactions of Xanthan and Sodium Stearoyl Lactylate. Food Chem..

[78] Jiang Y., Zhang C., Yuan J., Wu Y., Li F., Waterhouse G.I.N., Li D., Huang Q. (2021). Exploiting the Robust Network Structure of Zein/Low-Acyl Gellan Gum Nanocomplexes to Create Pickering Emulsion Gels with Favorable Properties. Food Chem..

[79] Aston R., Sewell K., Klein T., Lawrie G., Grøndahl L. (2016). Evaluation of the Impact of Freezing Preparation Techniques on the Characterisation of Alginate Hydrogels by Cryo-SEM. Eur. Polym. J..

[80] Reyntjens S., Kübel C. (2005). Scanning/Transmission Electron Microscopy and Dual-Beam Sample Preparation for the Analysis of Crystalline Materials. J. Cryst. Growth.

[81] Yang X., Gong T., Lu Y., Li A., Sun L., Guo Y. (2020). Compatibility of Sodium Alginate and Konjac Glucomannan and Their Applications in Fabricating Low-Fat Mayonnaise-like Emulsion Gels. Carbohydr. Polym..

[82] Xing X., Dan Y., Xu Z., Xiang L. (2022). Implications of Oxidative Stress in the Pathogenesis and Treatment of Hyperpigmentation Disorders. Oxidative Med. Cell. Longev..

[83] Kang S.K., Kim K., Jeong J., Hong S., Park Y., Shin J. (2024). In Silico Full-Angle High-Dynamic Range Scattering of Microscopic Objects Exploiting Holotomography. Biomed. Opt. Express.

[84] Xiao Y., Wei Q., Du L., Guo Z., Li Y. (2024). In Vitro Evaluation and in Situ Intestinal Absorption Characterisation of Paeoniflorin Nanoparticles in a Rat Model. RSC Adv..

[85] Martin-Piñero M.J., García M.C., Muñoz J., Alfaro-Rodriguez M.-C. (2019). Influence of the Welan Gum Biopolymer Concentration on the Rheological Properties, Droplet Size Distribution and Physical Stability of Thyme Oil/W Emulsions. Int. J. Biol. Macromol..

[86] Araújo E.A.M., Lima G.R., Santos De Melo L.A.D., Sousa L.B.D., Vasconcellos M.C.D., Conde N.C.D.O., Toda C., Hanan S.A., Alves Filho A.D.O., Bandeira M.F.C.L. (2021). Effect of a Copaiba Oil-Based Dental Biomodifier on the Inhibition of Metalloproteinase in Adhesive Restoration. Adv. Pharmacol. Pharm. Sci..

[87] Pochapski D.J., Carvalho Dos Santos C., Leite G.W., Pulcinelli S.H., Santilli C.V. (2021). Zeta Potential and Colloidal Stability Predictions for Inorganic Nanoparticle Dispersions: Effects of Experimental Conditions and Electrokinetic Models on the Interpretation of Results. Langmuir.

[88] Liu C., Teng Z., Lu Q.-Y., Zhao R.-Y., Yang X.-Q., Tang C.-H., Liao J.-M. (2011). Aggregation Kinetics and ζ-Potential of Soy Protein during Fractionation. Food Res. Int..

[89] Mao Y., McClements D.J. (2011). Modulation of Bulk Physicochemical Properties of Emulsions by Hetero-Aggregation of Oppositely Charged Protein-Coated Lipid Droplets. Food Hydrocoll..

[90] Wang Y., Li D., Wang L.-J., Li S.-J., Adhikari B. (2010). Effects of Drying Methods on the Functional Properties of Flaxseed Gum Powders. Carbohydr. Polym..

[91] Chung C., Degner B., McClements D.J. (2013). Controlled Biopolymer Phase Separation in Complex Food Matrices Containing Fat Droplets, Starch Granules, and Hydrocolloids. Food Res. Int..

[92] Limpisophon K., Ma X., Sagis L.M.C., Nonthakaew A., Hirunrattana P. (2024). Synergistic Effects of Alkaline and Heat Treatments on Structural and Functional Properties of Mung Bean Protein Isolate: Improving Physicochemical Stability of Plant-Based Emulsions. Int. J. Food Sci. Technol..

[93] Fan H., Meng L., Wang Y., Wu X., Liu S., Li Y., Kang W. (2011). Superior Thermal Stability Gel Emulsion Produced by Low Concentration Gemini Surfactant. Colloids Surf. A Physicochem. Eng. Asp..

[94] Parniakov O., Bals O., Barba F.J., Mykhailyk V., Lebovka N., Vorobiev E. (2016). Application of Differential Scanning Calorimetry to Estimate Quality and Nutritional Properties of Food Products. Crit. Rev. Food Sci. Nutr..

[95] Zhang X., Chen X., Gong Y., Li Z., Guo Y., Yu D., Pan M. (2021). Emulsion Gels Stabilized by Soybean Protein Isolate and Pectin: Effects of High Intensity Ultrasound on the Gel Properties, Stability and β-Carotene Digestive Characteristics. Ultrason. Sonochemistry.

[96] Jennings C.C., Devenport J.C., Marshall A., Kenealey J.D. (2025). Differential Scanning Calorimetry Quantification of Whey Proteins. J. Agric. Food Chem..

[97] Zhang M., Zhang B., Sun X., Liu Y., Yu Z., Wang X., Xu N. (2024). Freeze-Thaw Stability of Transglutaminase-Induced Soy Protein-Maltose Emulsion Gel: Focusing on Morphology, Texture Properties, and Rheological Characteristics. Int. J. Biol. Macromol..

[98] Luo Y., Cui H., Tang W., Fu Z., Pu C., Sun Q. (2025). Regulating Effect of Glycated Rice Bran Protein Aggregates and Resultant Emulsions on the Gelatinization and Retrogradation Properties of Rice Starch. Food Hydrocoll..

[99] Zhang J., Zhang W., Hao J., Li X., Xu D., Cao Y. (2022). In Vitro Digestion of Solid-in-Oil-in-Water Emulsions for Delivery of CaCO_3_. Food Hydrocoll..

[100] Galvão A.M.M.T., Costa G.F.D., Santos M.D., Pollonio M.A.R., Hubinger M.D. (2024). Replacing the Animal Fat in Bologna Sausages Using High Internal Phase Emulsion Stabilized with Lentil Protein Isolate (*Lens culinaris*). Meat Sci..

[101] Sompongse W., Hongviangjan W., Sakamut P. (2024). Interaction of Polysaccharides on Gel Characteristics and Protein Microstructure of Threadfin Bream Surimi Gel. Int. J. Food Sci. Technol..

[102] Liang B., Feng S., Zhang X., Ye Y., Sun C., Ji C., Li X. (2024). Physicochemical Properties and in Vitro Digestion Behavior of Emulsion Micro-Gels Stabilized by κ-Carrageenan and Whey Protein: Effects of Sodium Alginate Addition. Int. J. Biol. Macromol..

[103] Galić N., Dijanošić A., Kontrec D., Miljanić S. (2012). Structural Investigation of Aroylhydrazones in Dimethylsulphoxide/Water Mixtures. Spectrochim. Acta Part. A Mol. Biomol. Spectrosc..

[104] Feng S., Liu C., Liu Y., Yi S., Li J., Zhang B., Li X. (2025). Improving the Gel Properties of Nemipterus Virgatus Myosin Gel Using Soy Protein Isolate-Stabilized Pickering Emulsion. Food Chem..

[105] Feng S., Liu Y., Li J., Zhang B., Liu C., Li X. (2025). Mechanism of Improving Water-Holding Capacity of Nemipterus Virgatus Myosin Gel by Soy Protein Isolate-Stabilized Pickering Emulsion. LWT.

[106] Zhao Q., Zheng B., Li J., Cheong K.L., Li R., Chen J., Liu X., Jia X., Song B., Wang Z. (2024). Emulsion-Filled Surimi Gel: A Promising Approach for Enhancing Gel Properties, Water Holding Capacity, and Flavor. Trends Food Sci. Technol..

[107] Urbonaite V., Van Der Kaaij S., De Jongh H.H.J., Scholten E., Ako K., Van Der Linden E., Pouvreau L. (2016). Relation between Gel Stiffness and Water Holding for Coarse and Fine-Stranded Protein Gels. Food Hydrocoll..

[108] Zhao J., Chang B., Wen J., Fu Y., Luo Y., Wang J., Zhang Y., Sui X. (2025). Fabrication of Soy Protein Isolate-Konjac Glucomannan Emulsion Gels to Mimic the Texture, Rheological Behavior and in Vitro Digestion of Pork Fat. Food Chem..

[109] Xu Y., Huang L., Zhao Y., Jin F., Wang F. (2025). Walnut Glutenin Peptide-Based-Rhamnolipid Composite Emulsion Gels: Preparation, Characterization and Application as Margarine Alternatives. Food Chem..

[110] Zhang R., Ma Y., Lu Y., Gao Y., Mao L. (2024). Structural Responses of Zein-Based Oil-in-Glycerol Emulsion Gels during Freeze-Thawing and Heating. Colloids Surf. A Physicochem. Eng. Asp..

[111] Liu Y., Sun J., Wen Z., Wang J., Roopesh M.S., Pan D., Du L. (2024). Functionality Enhancement of Pea Protein Isolate through Cold Plasma Modification for 3D Printing Application. Food Res. Int..

[112] Baydin T., Arntsen S.W., Hattrem M.N., Draget K.I. (2022). Physical and Functional Properties of Plant-Based Pre-Emulsified Chewable Gels for the Oral Delivery of Nutraceuticals. Appl. Food Res..

[113] Hu Y., Wang L., Julian McClements D. (2024). Design, Characterization and Digestibility of β-Carotene-Loaded Emulsion System Stabilized by Whey Protein with Chitosan and Potato Starch Addition. Food Chem..

[114] Kozu H., Kobayashi I., Ichikawa S. (2025). A Review on In Vitro Evaluation of Chemical and Physical Digestion for Controlling Gastric Digestion of Food. Foods.

[115] Nie Y., Xiong Y.L., Jiang J. (2025). Texture, Microstructure, and in Vitro Digestion of Hybrid Meat Gel-Type Sausages Formulated with Functionalized Pea Protein. Food Hydrocoll..

[116] Cui C., Wei Z., Hong Z., Zong J., Li H., Peng C., Cai H., Hou R. (2023). Preparation of Water-in-Oil Pickering Emulsion Stabilized by Camellia Oleifera Seed Cake Protein and Its Application as EGCG Delivery System. LWT.

[117] Giacalone D., Bredie W.L.P., Frøst M.B. (2013). “All-In-One Test” (AI1): A Rapid and Easily Applicable Approach to Consumer Product Testing. Food Qual. Prefer..

[118] Badar I.H., Li Y., Liu H., Chen Q., Liu Q., Kong B. (2023). Effect of Vegetable Oil Hydrogel Emulsion as a Fat Substitute on the Physicochemical Properties, Fatty Acid Profile, and Color Stability of Modified Atmospheric Packaged Buffalo Burgers. Meat Sci..

[119] Li X., Zhou S., Chen H., Zhang R., Wang L. (2025). Pomelo Fiber-Stabilized Oil-in-Water Emulsion Gels: Fat Mimetic in Plant-Based Ice Cream. Food Bioprocess. Technol..

[120] Hu X., Xiang X., Cao M., Li S., McClements D.J. (2025). Plant-Based Marbled Salami Analogs: Emulsion-Loaded Microgels Embedded within Protein-Polysaccharide Hydrogel Matrices. Food Hydrocoll..

[121] Yan J., Liang X., Ma C., McClements D.J., Liu X., Liu F. (2021). Design and Characterization of Double-Cross-Linked Emulsion Gels Using Mixed Biopolymers: Zein and Sodium Alginate. Food Hydrocoll..

[122] Guo J., Gu X., Meng Z. (2024). Customized 3D Printing to Build Plant-Based Meats: Spirulina Platensis Protein-Based Pickering Emulsion Gels as Fat Analogs. Innov. Food Sci. Emerg. Technol..

[123] Wei W., Cui L., Meng Z. (2025). The Potential of Protein-Polysaccharide-Based O/W and W/O Emulsion Gels Strengthened by Solid Fat Crystallization as Realistic Fat Analogs. Food Chem..

[124] Kothuri V., Han J.H., Keum D.H., Kwon H.C., Kim D.H., Han S.G. (2025). Utilization of Emulsion Gels in Plant-Based Meat Analog Formulations: A Review. Food Hydrocoll..

[125] Shaghaghian S., McClements D.J., Khalesi M., Garcia-Vaquero M., Mirzapour-Kouhdasht A. (2022). Digestibility and Bioavailability of Plant-Based Proteins Intended for Use in Meat Analogues: A Review. Trends Food Sci. Technol..

[126] Fiorentini M., Kinchla A.J., Nolden A.A. (2020). Role of Sensory Evaluation in Consumer Acceptance of Plant-Based Meat Analogs and Meat Extenders: A Scoping Review. Foods.

[127] Penchalaraju M., Bosco S.J.D. (2022). Leveraging Indian Pulses for Plant-based Meat: Functional Properties and Development of Meatball Analogues. Int. J. Food Sci. Tech..

[128] Czapalay E.S., Dobson S., Marangoni A.G. (2025). Legume Starch and Flour-Based Emulsion Gels as Adipose Tissue Mimetics in Plant-Based Meat Products. Future Foods.

[129] Tahir A.B., Jiang B., Jingjing C., Ali K. (2024). Improving Functional Merit of Commercially Available Pea Protein Isolate by Employing a Combination of Physical, Chemical, and Enzymatic Modification. Food Biosci..

[130] Tahir A.B., Khalil A.A., Gull H., Ali K., AlMasoud N., Alomar T.S., Aït-Kaddour A., Aadil R.M. (2025). Enhancing Structural and Functional Properties of Commercially Available Pea Protein Isolate for Plant-Based Meat Analogues Using Combined pH-Shift, High-Intensity Ultrasound, and Heat Treatments. Ultrason. Sonochemistry.

[131] Domínguez R., Pateiro M., Munekata P.E.S., McClements D.J., Lorenzo J.M. (2021). Encapsulation of Bioactive Phytochemicals in Plant-Based Matrices and Application as Additives in Meat and Meat Products. Molecules.

[132] Tan Y., McClements D.J. (2021). Plant-Based Colloidal Delivery Systems for Bioactives. Molecules.

[133] Yerramathi B.B., Muniraj B.A., Kola M., Konidala K.K., Arthala P.K., Sharma T.S.K. (2023). Alginate Biopolymeric Structures: Versatile Carriers for Bioactive Compounds in Functional Foods and Nutraceutical Formulations: A Review. Int. J. Biol. Macromol..

[134] Muñoz-González I., Ruiz-Capillas C., Salvador M., Herrero A.M. (2021). Emulsion Gels as Delivery Systems for Phenolic Compounds: Nutritional, Technological and Structural Properties. Food Chem..

[135] Zhou Y., Zhu L., Li Y., Guo F., Chen L., Wang G., Shen Q., Liu X., Ding W. (2024). Encapsulation of Probiotics (Lactobacillus Plantarum) in Soyasaponin–Soybean Protein Isolate Water-in-Oil-in-Water (W/O/W) Emulsion for Improved Probiotic Survival in the Gastrointestinal Tract. LWT.

[136] Farooq S., Ijaz Ahmad M., Zhang Y., Chen M., Zhang H. (2022). Fabrication, Characterization and in Vitro Digestion of Camellia Oil Body Emulsion Gels Cross-Linked by Polyphenols. Food Chem..

[137] Jiang Z., Liao Q., Zhao Y., Ying R., Hayat K., Salamatullah A.M., Huang M. (2025). Development of Soy Protein Cold-Set Emulsion Gels for Vitamin D3 Vehiculation: Enhanced Properties, Stability, and Sustained Release. Food Biosci..

[138] Li B., Luan H., Qin J., Zong A., Liu L., Xu Z., Du F., Xu T. (2024). Effect of Soluble Dietary Fiber on Soy Protein Isolate Emulsion Gel Properties, Stability and Delivery of Vitamin D3. Int. J. Biol. Macromol..

[139] Abu-El Khair A.G., Soliman T.N., Hashim A.F. (2023). Development of Composite Nanoemulsion Gels as Carriers for Co-Delivery of Wheat Germ Oil and Probiotics and Their Incorporation in Yoghurt. Food Biosci..

[140] Zhu S., Stieger M.A., Van Der Goot A.J., Schutyser M.A.I. (2019). Extrusion-Based 3D Printing of Food Pastes: Correlating Rheological Properties with Printing Behaviour. Innov. Food Sci. Emerg. Technol..

[141] Mohammadi A., Kashi P.A., Kashiri M., Bagheri A., Chen J., Ettelaie R., Jäger H., Shahbazi M. (2023). Self-Assembly of Plant Polyphenols-Grafted Soy Proteins to Manufacture a Highly Stable Antioxidative Pickering Emulsion Gel for Direct-Ink-Write 3D Printing. Food Hydrocoll..

[142] Eveland R., Antloga K., Meyer A., Tuscano L. (2024). Low Temperature Vaporized Hydrogen Peroxide Sterilization of 3D Printed Devices. 3D Print Med..

[143] Yiu C.C.-Y., Liang S.W., Mukhtar K., Kim W., Wang Y., Selomulya C. (2023). Food Emulsion Gels from Plant-Based Ingredients: Formulation, Processing, and Potential Applications. Gels.

[144] Mefleh M., Pasqualone A., Caponio F., De Angelis D., Natrella G., Summo C., Faccia M. (2022). Spreadable Plant-based Cheese Analogue with Dry-fractioned Pea Protein and Inulin–Olive Oil Emulsion-filled Gel. J. Sci. Food Agric..

[145] Zhang H., Wei A., Zhou S., Zhang H., Xia N., Wang J., Ma Y., Fan M. (2024). Effect of the Substitution of Butter by Double Cross-Linked Egg Yolk Granules/Sodium Alginate Emulsion Gel on Properties of Baking Dough during Frozen Storage. Food Chem..

[146] Gutiérrez-Luna K., Astiasaran I., Ansorena D. (2023). Fat Reduced Cookies Using an Olive Oil-Alginate Gelled Emulsion: Sensory Properties, Storage Stability and in Vitro Digestion. Food Res. Int..

[147] Geng S., Liu X., Ma H., Liu B., Liang G. (2021). Multi-Scale Stabilization Mechanism of Pickering Emulsion Gels Based on Dihydromyricetin/High-Amylose Corn Starch Composite Particles. Food Chem..

